# Inhibition of poly (adenosine diphosphate-ribose) polymerase attenuates lung-kidney crosstalk induced by intratracheal lipopolysaccharide instillation in rats

**DOI:** 10.1186/1465-9921-14-126

**Published:** 2013-11-15

**Authors:** May Khin Hnin Si, Chieko Mitaka, Miniwan Tulafu, Shinya Abe, Masanobu Kitagawa, Satoshi Ikeda, Yoshinobu Eishi, Shunichi Kurata, Makoto Tomita

**Affiliations:** 1 Departments of Critical Care Medicine, Tokyo Medical and Dental University Graduate School, 1-5-45, Yushima, Bunkyo-ku, Tokyo 113-8519, Japan; 2 Comprehensive Pathology, Tokyo Medical and Dental University Graduate School, 1-5-45, Yushima, Bunkyo-ku, Tokyo 113-8519, Japan; 3 Human Pathology, Tokyo Medical and Dental University Graduate School, 1-5-45, Yushima, Bunkyo-ku, Tokyo 113-8519, Japan; 4 Redox Response Cell Biology, Tokyo Medical and Dental University Medical Research Institute, 1-5-45, Yushima, Bunkyo-ku, Tokyo 113-8519, Japan; 5 Clinical Research Center, Tokyo Medical and Dental University Hospital of Medicine, 1-5-45, Yushima, Bunkyo-ku, Tokyo 113-8519, Japan

**Keywords:** Acute respiratory distress syndrome (ARDS), 3-aminobenzamide (3-AB), Cytokines, Lipopolysaccharide, Nuclear factor (NF)-κB, Lung and kidney, Poly (adenosine-diphosphate ribose) polymerase (PARP)

## Abstract

**Background:**

Acute respiratory distress syndrome (ARDS) is a severe form of lung injury that frequently occurs during pneumonia and sepsis. Lung inflammation in ARDS patients may have deleterious effects on remote organs such as the kidney. The nuclear enzyme poly(adenosine diphosphate-ribose) polymerase (PARP) enhances the nuclear factor (NF)-κB-dependent transcription of inflammatory cytokines. This study was conducted to elucidate two questions: first, whether the activation of PARP and NF-κB mediates the renal inflammation secondary to the lipopolysaccharide (LPS)-induced acute lung inflammation; second, whether a PARP inhibitor, 3-aminobenzamide (3-AB), attenuates lung and kidney inflammation by inhibiting NF-κB-dependent proinflammatory cytokines.

**Methods:**

Male Sprague–Dawley rats were anesthetized, ventilated, and divided into three groups; a control group (n = 8); an LPS group (n = 12) intratracheally instilled with LPS (16 mg/kg), and an LPS + 3-AB group (n = 12) given the same dose of LPS by the same method followed by an intravenous injection of 3-AB (20 mg/kg). Hemodynamics, arterial blood gas, and the plasma levels of lactate, creatinine and potassium were measured at 0,1,2,3, and 4 h after treatment. The lung wet/dry ratio was measured at 4 h. The mRNA expression of tumor necrosis factor (TNF)-α, interleukin (IL)-1β and IL-6 in the lung and kidney were measured by TaqMan real-time PCR. PARP and NF-κB in the lung and kidney were histologically examined by immunostaining and assigned expression scores.

**Results:**

LPS induced metabolic acidosis, hypotension, hypoxemia, increased the lung wet/dry ratio, increased the plasma levels of creatinine and potassium, and increased the cytokine mRNA expressions in the lung and kidney. All of these effects were associated with strong expression of PARP and NF-κB. Treatment with 3-AB prevented the LPS-induced metabolic acidosis and hypotension, reduced the plasma levels of lactate, creatinine and potassium, reduced the cytokine mRNA expressions, reduced the expression of PARP and NF-κB, improved pulmonary edema and oxygenation and preserved renal function.

**Conclusions:**

The PARP inhibition attenuated lung-kidney crosstalk induced by intratracheal LPS instillation, partly via an inhibition of NF-κB dependent proinflammatory cytokines.

## Introduction

Acute respiratory distress syndrome (ARDS) is a severe form of lung injury and inflammation that frequently occurs during pneumonia and sepsis [1]. Endotoxin is a term currently used to describe lipopolysaccharide (LPS), an outer membrane component of Gram-negative bacteria that can cause severe inflammation by triggering the production of various proinflammatory cytokines [2]. The acute lung inflammation in septic lung injury may have deleterious effects on remote organs such as the kidney and may involve cross-talk between the lung and kidney [3]. Little is certain, however, regarding the responses of the kidney to acute lung inflammation or the mechanisms underlying those responses.

The enzyme poly (adenosine diphosphate-ribose) polymerase (PARP) catalyzes the attachment of ADP ribose units to target proteins and plays an important role in modulating both chromatin structure and transcription [4]. PARP has been shown to sense DNA damage, repair DNA, and maintain genomic stability [5]. Yet excessive PARP activation after massive DNA damage may aggravate inflammatory response. PARP has also been shown to enhance the nuclear factor (NF)-κB-dependent transcription of inflammatory cytokines [6-9]. Treatment with PARP inhibitors improves the pathogenesis of septic acute lung inflammation [10,11] and ventilator-induced lung injury [12]. As such, PARP inhibitor may ameliorate the inflammation of the kidney as well as the lung by blocking NF-κB dependent inflammatory cytokines. This study was conducted to clarify two questions: first, whether the activation of PARP and NF-κB mediates the renal inflammation secondary to lipopolysaccharide (LPS)-induced acute lung inflammation; second, whether a PARP inhibitor, 3-aminobenzamide (3-AB), attenuates lung and kidney inflammation by inhibiting NF-κB-dependent proinflammatory cytokines.

## Materials and methods

This study was approved by the Institutional Animal Care Committee of Tokyo Medical and Dental University. The care and handling of the animals were in accordance with the National Institute of Health guidelines. Male Sprague–Dawley rats (mean body weight 291.5 ± 3.4 g) were anesthetized with an intraperitoneal injection of pentobarbital sodium (5 mg/100 g body weight). After tracheostomy, the rats were mechanically ventilated with a rodent ventilator (SN-408-7 Respirator, Shinano Manufacturing Co. Ltd, Japan) under the following conditions: F_I_O_2_ 0.21, tidal volume of 10 ml/kg, 5 cmH_2_O positive end-expiratory pressure and respiratory rate of 30–40 cycles/min. The right carotid artery was cannulated with a catheter for continuous measurement of the arterial pressure and heart rate and for intermittent arterial blood samplings. The catheter was connected to a transducer, the transducer was calibrated to zero at the midchest, and pressure was measured with a blood pressure amplifier (AP-641G, SEN-6102 M, Nihon Kohden, Tokyo, Japan) and data acquisition system (Power Lab2/26, ML826, ADInstruments, Australia). The right femoral vein was cannulated with a catheter for intravenous injection of 3-AB and additional anesthetics.

### Experimental protocol

The rats were randomized to three groups: 1) a control group (n = 8); 2) an LPS group (n = 12) intratracheally instilled with LPS (*Escherichia coli* O111:B4, Sigma-Aldrich, Inc., St. Louis, MO, USA) at a dose of (16 mg/kg) in 0.5 ml normal saline via an intratracheal aerosolizer (PennCentury, Inc., Philadelphia, PA, USA); and 3) an LPS + 3-AB group (n = 12) given the same dose of LPS by the same method followed by an intravenous injection of 3-AB (Sigma-Aldrich, Inc., St.Louis, MO, USA) in 0.5 ml normal saline. The 3-AB given to the latter group was administered at a dose of 20 mg/kg, the same dose used in the experiments by Yu J, et al. [13]. According to the report from Yu, 3-AB at the 20 mg/kg dose attenuated PARP activation and was proven not to be toxic to the liver or kidney in rats. The hemodynamics, arterial blood gas, and plasma levels of lactate, creatinine and potassium were measured at 0, 1, 2, 3 and 4 h after treatment. Blood gas analysis was performed on a blood gas analyzer (ABL 837, Radiometer, Copenhagen, Denmark). The rats were killed with an overdose of pentobarbital upon completion of the experiment at 4 h, and the lung and kidney were harvested with a 10% formaldehyde solution at 4°C for histologic examination and preserved at −80°C until cytokine mRNA analysis.

### Wet/dry ratio of the lung

The wet/dry ratio of the lung was calculated as a parameter of lung edema by desiccating the lung at 80°C for 24 h.

### RNA extraction and TaqMan real-time PCR

Total RNA was extracted from the lung and kidney with TRIzol reagent (Invitrogen, Carlsbad, CA, USA) according to the manufacturer’s instruction. The RNA concentration was determined by the absorbance read at 260 nm (GeneQuant100, GE Healthcare UK Ltd, Buckinghamshire, UK). cDNA was synthesized using TaqMan reverse transcription reagents (Applied Biosystems, Roche Molecular Systems, Inc., NJ, USA) and quantified using a thermal cycler (PC707, ASTEC Co., Ltd., Japan). The primers and TaqMan probes for tumor necrosis factor (TNF)-α, interleukin (IL)-1β, IL-6 and glyceraldehyde-3-phosphate dehydrogenase (GAPDH) mRNA were purchased from a commercial laboratory (Applied Biosystems, Foster city, CA, USA). The mRNA expression of TNF-α, IL-1β and IL-6 were determined by TaqMan real-time PCR using an ABI 7900HT (Applied Biosystems, Foster City, CA, USA). TaqMan rat GAPDH was used as an internal control and relative gene expression values were determined using the 2 ^–ΔΔCT^ method [14].

### Histological examination of PARP and NF-κB in lung and kidney

Antibodies for PARP and NF-κB were used for immunostaining. The sections were deparaffinized by xylene. The latter immunostaining was performed by the following procedure. For PARP immunostaining, the sections were heat treated in citric acid buffer at pH 6.0 in a microwave oven for 20 minutes and then air-cooled for 20 minutes. For NF-κB immunostaining, the sections were treated with H_2_O_2_ for 10 minutes to inactivate the endogenous peroxidase. Next, anti PARP rabbit monoclonal antibody and anti NF-κB rabbit monoclonal antibody (Epitomics, Burlingame, CA, USA) (diluted 1:200) were added to the sections in a moisture chamber and reacted for 3 h at room temperature. After a 30-minutes wash in phosphate buffer solution, the sections were examined by the polymer method at room temperature using a Novo Link Polymer kit (Leica Microsystems).The linker and polymer in the kit were reacted for 30 minutes each and visualized in DAB. The samples were then counterstained by hematoxylin, dehydrated, and coverslipped. Adjacent sections were also examined with hematoxylin and eosin staining for conventional histopathologic examination.

The PARP and NF-κB expressions were scored by observing the positively stained areas and staining intensity of five randomly selected fields under high power magnification (×200) and applying semiquantitative scores. Scores of 3, 2, 1, and 0 were assigned to fields with strong, moderate, weak, and negligible staining for PARP and NF-κB, respectively. All of the judgments were performed under a blind condition to avoid bias.

### Statistical analysis

All data are shown as mean ± SE. The hemodynamics, blood gas variables, and plasma levels of lactate, creatinine, and potassium were analyzed by a Kruskal-Wallis test at a fixed time (4 h later in most experiments) since there were interactions of groups against time in repeated measure ANOVA for these data. The Kruskal-Wallis test was applied for comparison of the wet/dry ratio, cytokine mRNA expression, PARP scoring, and NF-κB scoring among the three groups. When the result from the Kruskal-Wallis test was significant, then Mann–Whitney U test was similarly applied within all two-group combinations. A *p* value of less than 0.05 was considered statistically significant.

## Results

### Changes in hemodynamics, the lung wet/dry ratio, and plasma levels of lactate, creatinine, and potassium

**Figure 1:**
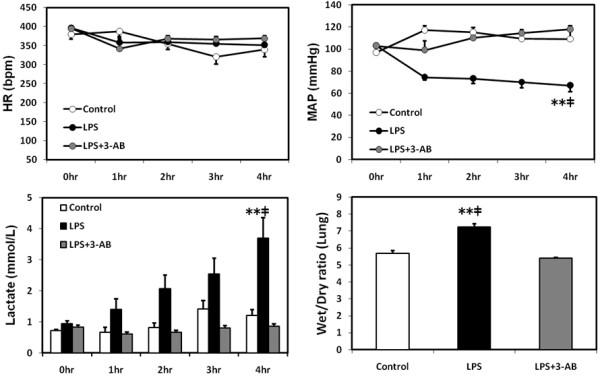
**Changes in the heart rate, mean arterial pressure, plasma lactate level and lung wet/dry ratio.** Values are expressed as mean ± SE. **p < 0.01 vs. the LPS + 3-AB group, ‡*p* < 0.01 vs. the control group.

No statistical differences in the heart rate were seen among the three groups. Intratracheal instillation of LPS induced a significant (*p* < 0.01) fall in mean arterial pressure compared with the control, and the administration of 3-AB attenuated the LPS-induced hypotension. The lung wet/dry ratio and plasma levels of lactate, creatinine, and potassium at 4 h were all significantly (*p* < 0.01) higher in the LPS group than in the control group or LPS + 3-AB group (lung wet/dry ratio, Figure 1; plasma creatinine, 0.22 ± 0.02 mg/dl vs. 0.17 ± 0.02 mg/dl and 0.11 ± 0.003 mg/dl, respectively; plasma potassium, 3.24 ± 0.28 mmol/L vs. 2.83 ± 0.14 mmol/L and 2.4 ± 0.05 mmol/L, respectively).

### Changes in arterial blood gas variables

**Figure 2:**
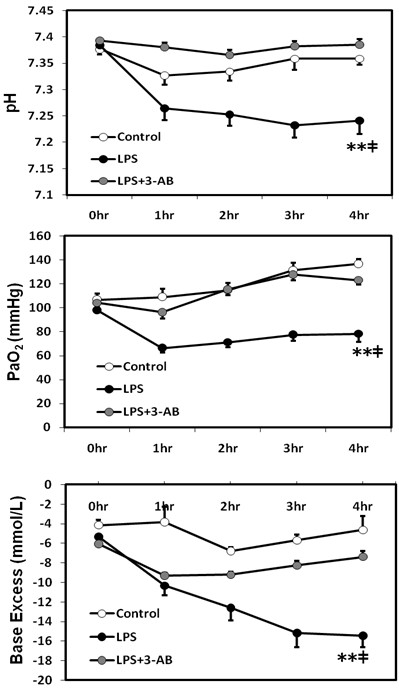
**Changes in arterial blood gas variables.** Values are expressed as mean ± SE. **p < 0.01 vs. the LPS + 3-AB group, ‡*p* < 0.01 vs. the control group.

The arterial blood gas pH at 4 h was significantly (*p* < 0.01) lower in the LPS group than in the LPS + 3-AB group or control group (Figure 2). The PaO_2_ at 4 h was significantly (*p* < 0.01) lower in the LPS group than in the control group or LPS + 3-AB group. No significant differences in PaCO_2_ were detected among the three groups. The base excess at 4 h was significantly (*p* < 0.01) lower in the LPS group than in the control group or LPS + 3-AB group (Figure 2).

### Cytokine mRNA expressions in lung and kidney

**Figure 3:**
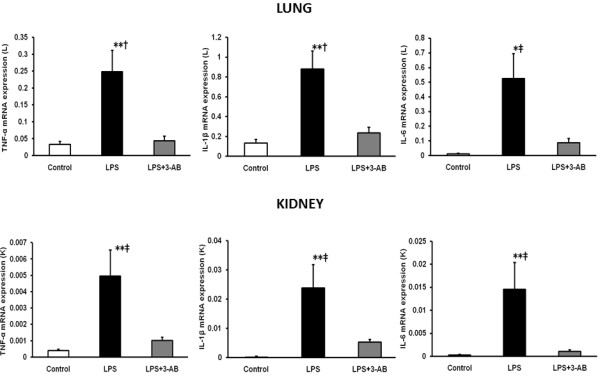
**Comparison of mRNA expression of cytokines in lung and kidney relative to GAPDH.** Total RNA was extracted from lung (L) and kidney (K) following 4 h after exposure to LPS and quantified relative to GAPDH by TaqMan real-time PCR. Each column with a bar represents the mean ± SE. *p < 0.05, **p < 0.01 vs. the LPS + 3-AB group, †*p* < 0.05, ‡*p* < 0.01 vs. the control group.

The mRNA expressions of TNF-α, IL-1β and IL-6 in the lung and kidney were significantly (*p* < 0.05) higher in the LPS group than in the control group or LPS + 3-AB group (Figure 3).

### Histopathological analysis in lung and kidney

Perivascular edema of the lung and kidney was observed in the LPS group, but not in the control group or LPS + 3-AB group. No evidence of inflammatory cell infiltration was observed in the lung or kidney of any of the three groups.

### Histological detection and localization of PARP and NF-κB in the lung and kidney

**Figure 4:**
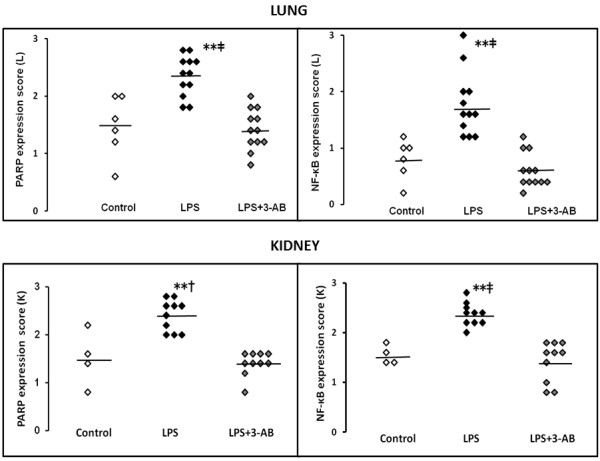
**Evaluation and comparison of PARP and NF-κB expression scores in lung and kidney.** PARP and NF-κB protein was stained in brown and an expression score was assigned by observing the staining intensity of five randomly selected fields under high power magnification (×200) and applying semiquantitative scores. Scores of 3, 2, 1, and 0 were assigned to fields with strong, moderate, weak, and negligible staining for PARP and NF-κB, respectively. **p < 0.01 vs. the LPS + 3-AB group, ‡*p* < 0.01 vs. the control group. †*p* < 0.05 vs. the control group. Line represents mean.

PARP and NF-κB were detected and localized in the bronchial epithelial cells of the lung and in the proximal tubules of the kidney. The expressions of PARP and NF-κB in the lung were significantly (*p* < 0.01) higher in the LPS group than in the control group or LPS + 3-AB group (Figure 4, upper panel). The expressions of PARP and NF-κB in the kidney were significantly (*p* < 0.05) higher in the LPS group than in the control group or LPS + 3-AB group (Figure 4, lower panel).

PARP and NF-κB proteins were strongly stained in the LPS group, weakly stained in the LPS + 3-AB group, and almost entirely unstained in the control group (Additional file 1).

## Discussion

The intratracheal instillation of LPS induced metabolic acidosis and hypotension, decreased arterial oxygenation, and increased the lung wet/dry ratio and mRNA expressions of TNF-α, IL-1β and IL-6 in the lung and kidney. In the animals additionally given the PARP inhibitor 3-AB, the PARP inhibitory effect improved arterial oxygenation and pulmonary edema and prevented LPS from inducing metabolic acidosis and hypotension or increasing the mRNA expressions of TNF-α, IL-1β and IL-6 in the lung and kidney. Histopathological analysis further revealed perivascular edema of the lung and kidney in the LPS group, but not in the control group or LPS + 3-AB group. The PARP inhibitor 3-AB thus seems to confer protective effects in reducing pulmonary edema and kidney edema. Taken together, these findings demonstrate the important role of PARP activation in the development of lung and kidney inflammation following the administration of LPS. The current study is also the first to demonstrate that LPS-induced lung-kidney crosstalk is related to proinflammatory cytokines, and that PARP inhibition attenuates the lung-kidney crosstalk partly via the modulation of NF-kB dependent proinflammatory cytokines.

The intratracheal administration of LPS initiates a lung inflammatory response [12,15-17] useful for preparing an animal model of acute lung injury and ARDS [18,19]. LPS has the potential to induce symptoms of sepsis, even when administered intratracheally [18]. DNA strand breakage activates PARP in states of endotoxemia and inflammatory response, and excessive activation induces cell dysfunction and cell death by depleting the cellular stores of adenosine triphosphate [4,5]. Pharmacological inhibition of PARP has been investigated in various experimental conditions of acute lung injury and LPS-induced organ injury [10,11,20-23]. Three-AB is a well-recognized competitive inhibitor of PARP that helps to reduce the degree of tissue injury caused by myocardial infarction [24] and laryngeal injury [25] in rats. Three-AB has also been shown to preserve mitochondrial respiration, NAD^+^, and ATP [26]. This preservative effect suggests that PARP inhibition may improve cellular energy homeostasis and prohibit cell dysfunction and death. Our results prove that 3-AB prevents metabolic acidosis, a typical sign of lactic acidosis and acute kidney injury. PARP inhibition is thus confirmed to preserve cellular energy homeostasis and maintain kidney function during inflammatory cell injury.

Our experiment was designed without a control group given 3-AB alone. We know, however, that 3-AB administration to rabbits brings about no changes in the mean arterial pressure, central venous pressure, pulmonary arterial pressure, cardiac output, or alveolar-arterial oxygen difference (AaDO_2_) after 4 h [20]. Pulmonary edema and hemodynamic changes thus seem unlikely to develop in response to 3-AB, as an increase of AaDO_2_ is associated with edema formation. Changes were similarly absent in the gene expressions of TNF-α, IL-1β, and IL-6 in lungs and in the number of PARP-positive epithelial cells in mice receiving 3-AB without intratracheal LPS administration [27]. Taken together, these findings suggest that 3-AB alone confers no effects on hemodynamics, pulmonary edema, cytokine gene expression or PARP activation.

The significantly high plasma creatinine and potassium levels, the biochemical parameters for renal function, at 4 h in our LPS group indicated that intratracheal LPS induced injury and inflammation not only in the lung, but also in the kidney via organ crosstalk between the lung and kidney. Along with this line, the LPS-treated rats exhibited significantly higher expressions of PARP and NF-κB in the lung and kidney and significantly higher mRNA expression of the NF-κB-dependent proinflammatory cytokines TNF-α, IL-1β and IL-6 in both organs. The enhanced expression levels partly implicate PARP activation as a cause of renal inflammation in LPS-induced lung inflammation and demonstrate PARP’s effect as a mediator of the transcriptional activation of NF-κB-dependent cytokines. The present study focused little on meaningful specific targets of downstream NF-κB signaling, but earlier studies have shown the LPS-induced NF-κB signaling pathway through PARP activation. LPS enhanced the binding of PARP-1 with the NF-κB subunit p65 (RelA) and poly(adenosine diphosphate-ribosyl)ation of p65, which in turn upregulated the transcriptional activity of the NF-κB and mRNA expressions of IL-1β in murine macrophages [28]. The extracellular signal-regulated kinase (ERK)-dependent phosphorylation of PARP-1 also regulated PARP-1 activity and NF-κB activation [28]. PARP inhibitor activated the phosphatidylinositol 3-kinase/AKT pathway and inactivated the ERK1/2and p38 mitogen-activated protein kinase in LPS-induced inflammation in mice, which resulted in the inactivation of NF-κB [29].

Renal dysfunction occurs as a consequence of ventilator-induced lung injury superimposed with LPS via the peroxynitrite-induced PARP activation, and pretreatment with PARP inhibitors confers beneficial effects on lung and kidney injury [12,17]. Moreover, PARP over-activation has been observed in acute renal dysfunction induced by LPS, and PARP inhibition has been identified as a potential target for AKI caused by LPS [23].

The renal inflammation secondary to LPS-induced acute lung inflammation was mediated via the activation of PARP and NF-κB in the present study. Regarding lung-kidney crosstalk, acute lung inflammation and the associated mechanical ventilation induced biotrauma by releasing proinflammatory cytokines into the systemic circulation and distant organs such as the kidney. Furthermore, acute lung inflammation with subsequent blood gas changes had adverse effects on renal hemodynamics and function. Treatment with 3-AB, a pharmacological inhibitor of PARP, attenuated the lung and kidney inflammation by inhibiting NF-κB-dependent proinflammatory cytokines. The 3-AB treatment appeared to promptly block the initiation of the vicious cycle between the lung and kidney. We thus conclude that 3-AB attenuates lung-kidney crosstalk, one of the mechanisms of multiple organ dysfunction syndrome.

No measurements of plasma endotoxin levels were taken in the present study. Another study has shown, however, that pulmonary-to-systemic translocation of endotoxin can occur [30]. Specifically, plasma endotoxin levels were significantly increased over a period from 40 min to 3 h after intratracheal instillation of LPS (500 μg) in mechanically ventilated rabbits [30]. In our study we administered a larger dose of LPS (16 mg/kg) intratracheally. Hence, the lung-to-kidney combined response in our animals may have been due to LPS escaped into the pulmonary circulation together with a secondary response by the kidney to soluble mediators released by the distressed lung.

PARP also takes part in the regulation of the expression of various proteins implicated in inflammatory and immune responses at the transcriptional level. NF-κB, the key transcriptional factor, plays a critical role in the regulation of these proteins, while PARP has been shown to act as a co-activator in the NF-κB mediated transcription [6,8]. The functional association between PARP-1 and NF-κB has been observed in association with the transcriptional activation of NF-κB and a systemic inflammatory response [7]. LPS induced PARP and NF-κB staining in the lungs and kidneys of our animals, and PARP inhibition markedly attenuated NF-κB staining in both organs. This result is consistent with the LPS-conferred rises in the mRNA expressions of TNF-α, IL-1β and IL-6 in these organs and the action of PARP inhibitor in attenuating the same. The lung PARP staining was relatively strong in the present study, while the kidney PARP staining was relatively weak. The lung NF-κB staining, meanwhile, was relatively weak, while the kidney NF-κB was relatively strong. We have no clear explanation for the different staining intensity between the lung and kidney. Many factors can influence the intensity of positive signals during color development with DAB solution. To control for this, we stained samples from each organ at the same time under identical conditions. The difference in staining intensity between the two organs may have been caused by a discrepancy in the time performance of the immunostaining between the organs. The difference is very unlikely to have stemmed from a translational component.

Three-AB competitively inhibits PARP by blocking PARP’s ability to ribosylate adenosine diphosphate without affecting its enzymatic (DNA-binding) activity. We administered 3-AB after LPS instillation in the present study, hence the expression of PARP might have been upregulated in the lung and kidney after the LPS-treatment. In experiments with rat astroglial cell cultures, the time course of PARP expression showed an up-regulation (about +90%) after only 1 h of LPS and interferon γ treatment and a progressive decrease hours later (18 h) [31]. We thus speculate that 3-AB may not have totally blocked PARP activity in the present study. We believe, instead, that 3-AB acted as a blockade of PARP transcription. Overall, our findings suggest that the PARP inhibitor 3-AB reduces lung and renal inflammation by inhibiting NF-κB stimulation.

## Conclusions

PARP plays an important role in LPS-induced lung and kidney inflammation. The inhibition of PARP by 3-AB markedly attenuates metabolic acidosis, biochemical derangements, and histological changes in the lung-kidney crosstalk encountered after intratracheal LPS instillation, partly via an inhibition of NF-κB-dependent proinflammatory cytokines.

## Competing interests

The authors declare that they have no competing interests.

## Authors’ contributions

MS and CM designed and carried out the experiment, acquisition of data and wrote the manuscript. MT, SA and MK took partial responsibility of real time PCR. SI, YE and SK participated and conducted immunohistochemical examination. MT performed the analysis. All authors read and approved the final manuscript.

## Supplementary Material

Additional file 1 **Representative immunostaining images of PARP and NF-κB in lung and kidney.** PARP and NF-κB proteins were immunostained in brown and showed strong staining in the bronchial epithelial cells of the lung (A) and in the proximal tubules of the kidney (B) in the LPS group. In contrast, PARP and NF-κB staining in the lung and kidney was weak in the LPS+3-AB group. The control group had a negligible staining. Click here for file
