# Supplementary figures and images for: Inhibition of poly (adenosine diphosphate-ribose) polymerase attenuates lung-kidney crosstalk induced by intratracheal lipopolysaccharide instillation in rats
Source: Respir Res. 2013 Nov 15;14(1):126. doi: 10.1186/1465-9921-14-126 (PMC3833186; doi:10.1186/1465-9921-14-126)

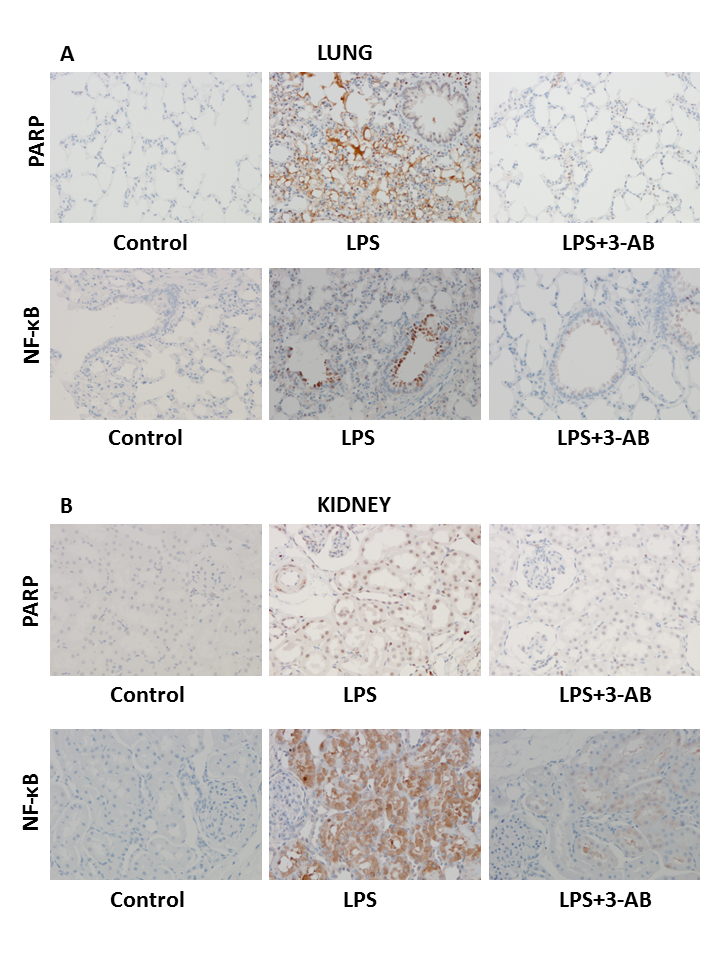

Supplement: Additional file 1 — Representative immunostaining images of PARP and NF-κB in lung and kidney. PARP and NF-κB proteins were immunostained in brown and showed strong staining in the bronchial epithelial cells of the lung (A) and in the proximal tubules of the kidney (B) in the LPS group. In contrast, PARP and NF-κB staining in the lung and kidney was weak in the LPS+3-AB group. The control group had a negligible staining. [file 1465-9921-14-126-S1.tiff]
